# Education and non-communicable diseases in India: an exploration of gendered heterogeneous relationships

**DOI:** 10.1093/inthealth/ihae037

**Published:** 2024-05-24

**Authors:** Jhumki Kundu, Srinivas Goli, K S James

**Affiliations:** Centre for Ageing Studies, International Institute for Population Sciences, Mumbai, Maharashtra 400088, India; Department of Fertility and Social Demography, International Institute for Population Sciences, Mumbai, Maharashtra 400088, India; Tulane University, New Orleans, LA, USA

**Keywords:** CVDs, diabetes, education, gender, India, NCDs

## Abstract

**Background:**

While the association between education and non-communicable diseases (NCDs) is well established, it remains unclear whether this association varies by gender. The aim of this study was to examine two critical research questions: whether the association of education and NCDs is conditioned by gender and, if so, what are the factors contributing to this?

**Methods:**

Data from the Longitudinal Aging Study in India Wave 1 (2017–2018) was used for the empirical analysis. The study employs bivariate, binary logistic regression and Oaxaca decomposition analyses.

**Results:**

The results reveal that the net likelihood of having at least one chronic NCD increases with an increase in education level for men (<5 y of schooling: odds ratio [OR] 1.18 [95% confidence interval {CI} 1.09 to 1.28]; ≥10 y of schooling: OR 1.43 [95% CI 1.33 to 1.53]). However, for women, the result showed a contrasting pattern. The decomposition analysis revealed that the distinctive roles of marital status and working status in the diagnosis of morbidity for men and women are the key factors behind the gendered heterogeneous relationship of education and NCDs in India.

**Conclusions:**

The study found that it is important to acknowledge the potential impact of self-reporting bias in morbidity data while examining the relationship between education and NCDs.

## Introduction

The unequal distribution of health around the world and across communities is an established fact.^1^ In his famous Social Determinants of Health (SDH) framework, Marmot says the social determinants are pertinent to both communicable and non-communicable diseases (NCDs) alike.^1^ A bourgeoning number of studies on social determinants of health have documented that health outcomes are influenced by an array of social factors beyond the realm of healthcare.^2^ The notable disparities in morbidity, mortality and risk factors, extensively documented by researchers within and across countries align with the fundamentals of the SDH framework.^3,4^ In particular, the evidence from both high-income and low-income countries highlights the importance of different axes of social power and health inequality and the pathways in which they influence health and healthcare. Thus a drive towards global health equity depends on research that precisely identifies and measures the evidence and draws pathways of how social determinants influence health.^5^

While education is a key human capital endowment that has a bearing on several socio-economic indicators, health is highly determined by the educational status of individual and families. However, within the traditional literature that continues to exert significant influence on our understanding of the impact of social inequality on health, different dimensions of inequality are typically perceived as a single axis.^6^ However, women bear additional layers of disadvantage and discrimination within the broad social parameters. For instance, ‘gender’ and ‘education’, two key social forces, have a significant bearing on health independently, but education may not be as advantageous as it for men due to intersections of additional layers of disadvantage and discrimination that women bear despite being educated.^7^ A few studies have found that gender and education often work together and interact with each other to determine health.^7–9^

However, in the absence of strong empirical evidence from developing countries, specifically from India, it is unclear to what extent the magnitude of the association of education and health, particularly concerning NCDs, is moderated by gender; for instance, how the association of education and NCDs are sensitive to gender roles and social hierarchies specific to gender status. More specifically, the evidence on how education and gender intersections operate in determining the health inequality and reporting of illness is relatively scarce. In subsequent sections, we elaborate on these linkages with existing literature and identify the gaps that need investigation.

### Education and health

Education as a social determinant of health is well established in public health research.^4^ Education and health are intricately linked at the core of individual and population health and well-being. While poor education is linked to poor health outcomes attributed to factors such as health knowledge, health-related behaviours, neighbourhood conditions and other socio-economic elements, poor health, in turn, is associated with educational challenges, including obstacles to learning due to disabilities, absenteeism or cognitive disorders.^10^

Enhancing education positively impacts health by fostering a greater sense of personal agency, which in turn motivates and empowers individuals to adopt and maintain a healthy lifestyle.^11^ Moreover, the positive effects of education on improving health are pervasive, cumulative and self-amplifying, increasing over the course of a person's life.^12^ Education also helps in better reporting of diseases, as educated individuals are more likely to have a better knowledge of diseases.^13^

Empirical evidence from numerous studies shows an ‘educational gradient in health,’ indicating a positive correlation between higher levels of schooling and improved health, as well as increased longevity. A pivotal work by Kitagawa and Hauser in 1973 highlighted significant mortality differences by education in the USA,^14^ a finding supported by subsequent research.^15,16^ Over the decades, various health outcomes, including general health, chronic conditions and functional limitations, continue to show strong associations with education. A majority of the studies that examined the association between education and NCDs in developing countries suggest there is a higher concentration of NCDs among educated individuals,^17,18^ which is in contrast to what was observed in the context of developed countries.^19,20^

### Gender and health

Gender, when considered as a factor influencing health, encompasses various interconnected aspects, including biological distinctions, psychological variations and societal encounters. The health status of both women and men results from the interplay between social (gender) and biological (sex) factors. Biological disparities in sex encompass factors such as the higher survival rates of female infants and the longer life expectancy of women. While women exhibit certain biological advantages in these aspects, any benefits they gain from these biological factors are offset by the social disadvantages they face.^21^

An expanding body of research has delved into gender disparity in health and mortality, commonly referred to as the ‘male–female health-survival paradox’.^22,23^ A substantial body of evidence affirms that women tend to outlive men on a global scale.^24,25^ Nevertheless, in North and Latin American and Caribbean nations,^26,27^ along with a few South Asian countries,^28,29^ there is a tendency for women to report poorer self-rated health and a higher prevalence and incidence of disability and chronic health issues compared with men. This phenomenon can be explained by two main factors. The first explanation, known as the biological perspective, emphasizes that hormonal and genetic differences between men and women are primarily responsible for this disparity. The second explanation, referred to as the psychological perspective, proposes that women's social roles and their attitudes toward illness play a significant role in the observed gender differences in morbidity.^30^

### Gender, health and education

In the realm of public health, the intertwined dynamics of education, gender and health have emerged as critical determinants shaping individual well-being. While the impacts of education and gender on health have been extensively studied, the interplay of gender, education and health remains relatively uncharted territory, particularly in the context of NCDs in developing countries. Some studies from developed countries have found a strong negative relationship between education levels and the probability of death from respiratory problems and accidents for men,^8,9^ while others report that education levels show a strong negative relationship with the prevalence of cardiovascular and cerebrovascular diseases in women.^8,9^ However, there are few studies in India that examine the gendered heterogeneous relationship between education and NCDs. In this context, this study aims to examine two critical research questions: whether the association of education and NCDs is conditioned by gender and, if so, what are the factors contributing to this?

## Methods

### Data source

The data used in this study were from wave 1 (2017–2018) of the Longitudinal Ageing Study in India (LASI). LASI wave 1 is collaborative effort by the International Institute for Population Sciences (IIPS), Harvard T.H. Chan School of Public Health (HSPH) and the University of Southern California (USC).

LASI is a nationally representative survey of the population ≥45 y of age in India and its states and union territories. The survey collects information on diseases, functional health, healthcare and the social and economic profiles of older adults based on internationally comparable measures.

**Figure 1.**
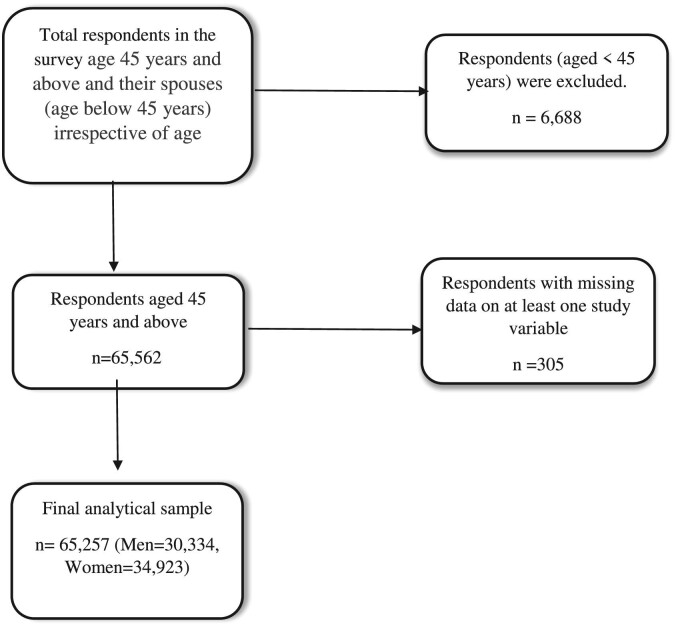
Sample selection criteria.

The LASI adopted a multistage stratified area probability cluster sampling design. The survey was conducted in a sample of 42 949 households and 72 250 individuals ≥45 y of age and their spouses, irrespective of age, across all states and union territories of India at the baseline. However, considering the empirical approach of this study, we restricted the samples of both respondents and their spouses to ≥45 y of age. Further, there were some missing observations in a few selected variables. Thus, after excluding both <45 y of age among spouses of the respondents and missing cases, the final analytical sample was 65 257 individuals ≥45 y of age (men 30 334, women 34 923) (Figure 1).

### Study variables

#### Outcome variables

The main outcomes variables were at least one NCD, cardiovascular disease (CVD) or diabetes. The presence of at least one NCD was derived from a direct question in the LASI survey: ‘Has any health professional ever diagnosed you with the following chronic conditions or diseases? including hypertension or high blood pressure; diabetes or high blood sugar; cancer or a malignant tumour; chronic lung disease such as asthma, chronic obstructive pulmonary disease/chronic bronchitis or other chronic lung problems; chronic heart diseases such as coronary heart disease (heart attack or myocardial infarction), congestive heart failure or other chronic heart problems; stroke; arthritis or rheumatism; osteoporosis or other bone/joint diseases and any neurological or psychiatric problems such as depression, Alzheimer's/dementia, unipolar/bipolar disorders, convulsions, Parkinson's etc.

CVDs were defined as any one of the self-reported diagnosed chronic conditions including hypertension or high blood pressure, chronic heart diseases such as coronary heart disease (heart attack or myocardial infarction), congestive heart failure, other chronic heart problems and stroke.

#### Key explanatory variable

Level of education is the main explanatory variable. The level of education of the respondent was categorized as no education, <5 y of schooling, 5–9 y of schooling and ≥10 y of schooling.

#### Socio-economic and demographic covariates

We controlled for all relevant socio-economic and demographic covariates to find the net association of the level of education and NCDs for the disaggregated samples of men and women. The covariates were selected based on the review of previous literature.^31,32^ These variables consisted of the respondent's age group, place of residence, religion, caste, marital status, living arrangement, working status, monthly per capita expenditure (MPCE) and region. Age was categorized into four groups: 45–49, 50–54, 55–59 and ≥60 y; place of residence was categorized as rural and urban; religion was categorized as Hindu, Muslim, Christian and other; caste was categorized as scheduled caste (SC)/scheduled tribe (ST), other backward class (OBC) and other; marital status was categorized as currently married, widowed and other (never married, divorced, separated, live-in relationship); living arrangement was categorized as living alone, living with a spouse and children and living with children and others; working status was categorized as never worked, ever worked but currently not working and currently working; the MPCE quintile was categorized as poorest, poorer, middle, richer and richest; and region was categorized as North, Central, East, West, North-east and South.

#### Statistical analyses

The analyses were conducted in four stages. First, descriptive statistics were estimated to show the distribution of the sample across the categories of the variables used in this study. Second, the prevalence of NCDs, CVDs and diabetes were estimated by education levels of women and men. Third, the adjusted prevalence of NCDs, CVDs and diabetes were estimated by education levels for women and men using predicted probability estimates from binary logistic regression. The logistic regression model was stratified by gender (men and women).

The mathematical formulation of the logistic regression model is as follows:


\begin{eqnarray*}
{\mathrm{Logit}}\left( {{\mathrm{NCDs}}} \right) &=& \ln \left( {{\mathrm{NCDs}}/1 - {\mathrm{\ NCDs}}} \right) = {\mathrm{\ \alpha }} + {{{\mathrm{\beta }}}_{\mathrm{1}}}{\mathrm{*}}{{{\mathrm{\chi }}}_{1\left( {{\mathrm{edu}}} \right)}}\\
&& +\, {{{\mathrm{\beta }}}_2}{\mathrm{*}}{{{\mathrm{\chi }}}_{2\left( {{\mathrm{SES\ Variables}}} \right)}}
\end{eqnarray*}

Fourth, we decomposed the differences in the prevalence of NCDs, CVDs and diabetes between the disadvantaged non-educated older adults and the advantageous educated older adults separately for men and women by employing a linear Blinder–Oaxaca decomposition model.^33^

The Blinder–Oaxaca linear decomposition model was used to decompose the contribution of various factors to the prevalence of NCDs, CVDs and diabetes. As this method is appropriate for binary outcome variables, we computed a binary variable of the level of education as disadvantageous older adults who have <10 y of schooling (coded 1) and advantageous older adults who have ≥10 y of schooling (coded 0). Our outcome variables are *y* (at least one NCD), *y*_1_ (CVDs) and *y*_2_ (diabetes). The gap between the mean outcome is as follows:


\begin{eqnarray*}
{{y}^{non \hbox{-} edu\ }}and\ {{y}^{\ edu}} &=& {{y}^{\ non \hbox{-} edu}} - \ {{y}^{edu}}\\
&=& \beta {{\ }^{non \hbox{-} edu\ }}{{x}^{non \hbox{-} edu}} -\, {{\beta }^{edu}}{{x}^{edu}},\end{eqnarray*}

where ${{x}^{non - edu}}$ and ${{x}^{edu}}$ are vectors of explanatory variables evaluated at the means of non-educated (<10 y of schooling) and educated (≥10 y of schooling), respectively.

Further, we estimated how much of the overall gap or the gap specific to any one of the *x*s is attributable to differences in *x*s (also called the explained component) and differences in *β*s (also called the unexplained component). Mathematically, it is expressed as follows:


\begin{eqnarray*}
{{y}^{non \hbox{-} edu}} - \ {{y}^{edu}} = \ \Delta x{{\beta }^{non \hbox{-} edu}} + \ \Delta \beta {{x}^{edu}}\end{eqnarray*}

where Δχ = χ*^edu^*−*χ^non-edu^* (explained component) and Δ*β* = *β^edu^*−*β^non-edu^* (unexplained component).

For the purpose of the Blinder–Oaxaca linear decomposition model^33^ we dichotomized socio-economic and demographic variables to perform the differential decomposition analysis: e.g. age group <60 y and ≥60 y, place of residence as rural and urban, marital status as widowed/other (never married, divorced, separated, live-in relationship) and currently married, religion as Muslim and other (Hindu, Christian and other), caste as SC/ST and other (OBC and other), working status as working and not working, living arrangement as living alone and living with spouse/children/others, economic status as poor and non-poor and region as North-east and other (North, East, West, Central and South).

## Results

Table 1 provides descriptive statistics of the variables used in this study. This study utilized information from a total of 65 257 older adults (46.5% men, 53.5% women). More than half of the older adults had never received any education, with a higher percentage of illiteracy among women (64.9%) than men (50.8%). A total of 10.9% of older adults had <5 y of schooling, 20.5% completed 5–9 y of schooling and 17.9% had ≥10 y of schooling. The percentage of women with higher education was significantly lower than that of men. Nearly half (46.2%) of the older adults were ever diagnosed with any chronic NCDs (46.2%) and the majority of (48.8%) them were women. A total of 29.5% of the older adults reported that they were diagnosed with CVD and among these 26.1% were men and 32.4% were women. A total of 12.3% of older adults reported having diabetes, with nearly equal numbers of men (12.5%) and women (12.2%). Complete details of the socio-economic and demographic profiles of the study sample are provided in Supplementary Table S1.

**Table 1.** Key features of the study sample

	Total population (N = 65 257)		Men (n = 30 334)		Women (n = 34 923)		
Variables	n	%	n	%	n	%	p-Value
Education level							
No education	30 687	50.8	9443	34	21 244	64.9	0.000
<5 y	7440	10.9	4042	13.3	3398	8.9	
5–9 y	14 803	20.5	8542	26.4	3398	15.4	
≥10 y	12 327	17.9	8307	26.3	4020	10.8	
NCD ever diagnosed							
Yes	30 168	46.2	13 006	43.1	17 162	48.8	0.000
No	35 089	53.8	17 328	56.9	17 761	51.2	
CVD ever diagnosed							
Yes	20 049	29.5	8282	26.1	11 767	32.4	0.000
No	45 208	70.5	22 052	73.9	23 156	67.6	
Diabetes ever diagnosed							
Yes	8401	12.3	4034	12.5	4367	12.2	0.000
No	56 856	87.7	26 300	87.5	30 556	87.8	

### Prevalence of NCDs by education level

**Figure 2.**
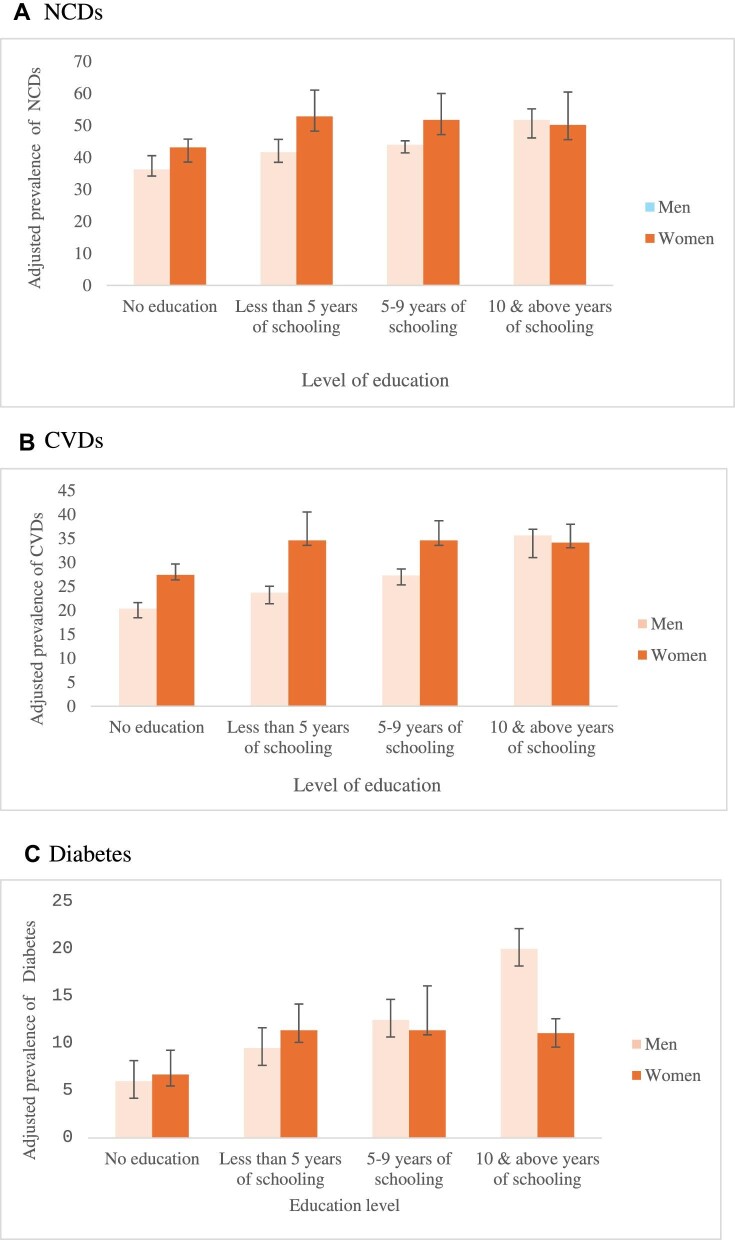
Adjusted prevalence of NCDs, CVDs and diabetes in older adults by education level in India, 2017–2018.

Figure 2 shows that the education gradient in NCD prevalence is sensitive to gender roles. After controlling for all sociodemographic and economic factors, the adjusted prevalence of NCDs among men consistently showed an upward trend with an increasing level of education: 36.2% (95% CI 34.1 to 40.5) in no education to 51.7% (95% CI 46.1 to 55.1) in ≥10 y of schooling. For women, education shows an inverted U-shape relationship with NCD prevalence: NCDs are low for non-educated women (43.1% [95% CI 42.3 to 45.7]), higher for middle educated women who have<5 y of schooling (52.8% [95% CI 51.9 to 58.1]) and then decline for women of with 5–9 y and ≥10 y of schooling (51.1% [95% CI 48.4 to 56.5]). The same pattern was observed in the case of CVDs and diabetes (Supplementary Table S2).

### Association of the prevalence of NCDs, CVDs and diabetes with education level

**Figure 3.**
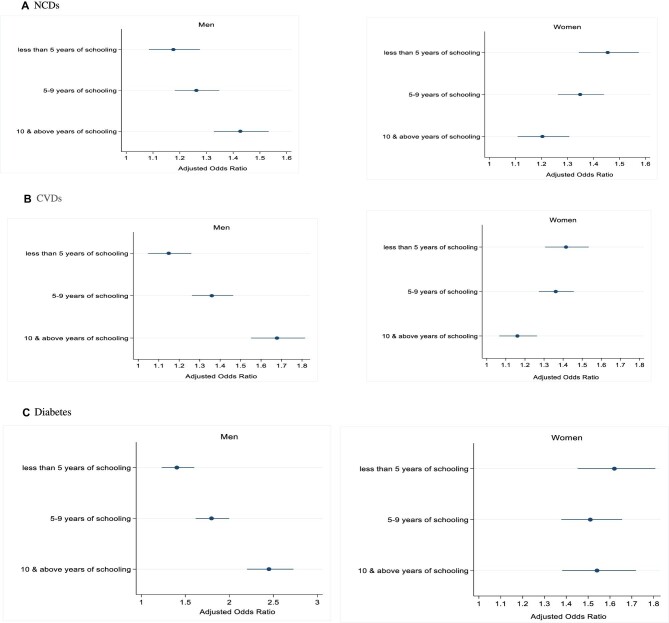
Odds of having NCDs, CVDs and diabetes by education level in older adults in India, 2017–2018.

In Figure 3, the odds of the prevalence of NCDs, CVDs and diabetes in older adults are depicted based on the education level by gender. Figure 3 illustrates that among men, compared with those with no education, the likelihood of having at least one NCD was 1.2 times higher (OR 1.18 [95% CI 1.09 to 1.28]) among those with <5 y of schooling, while it was 1.3 times (OR 1.26 [95% CI 1.18 to 1.35]) and 1.4 times higher (OR 1.43 [95% CI 1.33 to 1.53]) among those with 5–9 y of schooling and ≥10 y of schooling, respectively. Similarly, with reference to those with no education, the likelihood of having CVDs and diabetes was 1.2 times (OR 1.15 [95% CI 1.05 to 1.26]) and 1.4 times higher (OR 1.40 [95% CI 1.23 to 1.60]) for those with <5 y of schooling and 1.4 times (OR 1.36 [95% CI 1.26 to 1.46]) and 1.8 times higher (OR 1.69 [95% CI 1.47 to 1.93]) for those with 5–9 y of schooling. The highest prevalence of CVDs and diabetes was among those with ≥10 y of schooling, which was 1.7 times (OR 1.68 [95% CI 1.55 to 1.82) and 2.5 times higher (OR 2.45 [95% CI 2.20 to 2.73]) compared with those with no education.

However, the results revealed a contrasting pattern for women. The odds of occurrence of at least one NCD are low for non-educated women, increased for middle educated women who have <5 y of schooling (OR 1.46 [95% CI 1.34 to 1.57]) and then declined for women with 5–9 y of schooling (OR 1.35 [95% CI 1.26 to 1.44]) and ≥10 y of schooling (OR 1.20 [95% CI 1.11 to 1.31]). This same trend is also evident in the prevalence of CVDs and diabetes among women.

Supplementary Table S3 provides additional comprehensive details of the logistic regression models, elucidating the relationships between the prevalence of NCDs, CVDs and diabetes and the level of education, as well as other socio-economic variables stratified by gender.

### Mechanisms of gender heterogeneous education and health relationships

#### Decomposition analysis

Table 2 illustrates the results of the Blinder–Oaxaca decomposition analysis, revealing the relative proportional contributions of selected socio-economic and demographic factors to the gap in the prevalence of NCDs, CVDs and diabetes between educated (≥10 y of schooling) and non-/less-educated (<10 y of schooling) individuals. The results are presented separately for men and women.

**Table 2.** Oaxaca decomposition. Contribution of selected predictors to the differences in NCDs, CVDs and diabetes prevalence between educated and non-educated older adults by gender in India, 2017–2018

	Summary of Oaxaca decomposition					
	NCDs		CVDs		Diabetes	
Co-variates (% contribution of explanatory factors to total difference)	Men	Women	Men	Women	Men	Women
Age group	−16.8***	−28.8***	−16.2***	−35.8***	−8.6***	−14.4***
Place of residence	55.0***	71.5***	63.8***	81.0***	68.0***	75.9***
Living arrangement	0.1	0.7	0.1	1.5	0.1	0.1
Religion	−2.9***	−5.4***	−2.9***	−8.2***	−1.2	−2.7***
Caste	16.7***	18.6***	13.2***	19.2***	8.7***	7.6***
Marital status	3.9***	−11.7***	3.2***	−15.8***	4.0***	1.7
Working status	3.2	13.6***	3.4*	14.8***	1.7	8.7***
MPCE quintile	37.6***	40.9***	35.3***	43.2***	25.4***	22.9***
Region	3.0***	0.6	0.2	0.1	1.9***	0.2
% explained	55.4	84	38.8	95.6	38.2	55.7
% unexplained (residual)	44.6	16	61.2	4.4	61.8	44.3

***p<0.01, ** p<0.05, *p<0.1.

This table is derived from Supplementary Table S3.

Among men, the socio-economic and demographic predictors explain around 55% of the overall differences in the prevalence of NCDs between educated and non-educated individuals. Among women, these factors explained about 84% of the total differences in the prevalence of NCDs between educated and non-educated individuals. For CVD prevalence, the socio-economic and demographic factors contributed around 39% of the overall educational differences among men and 84% among women. Similarly, the same factors contributed 38% and 56%, respectively, in men's and women's educational differences in diabetes prevalence.

Further, the results demonstrate that the place of residence was the largest contributor to the educational gap in the prevalence of NCDs among both men (55%) and women (71.5%), which is followed by economic status and caste. This pattern was consistent for both CVDs and diabetes as well. The large contribution from the place of residence clearly indicates gaps in availability and access to medical care and facilities between rural and urban areas, contributing to the difference in diagnosis and reporting of morbidities, especially among lower-educated people.

**Figure 4.**
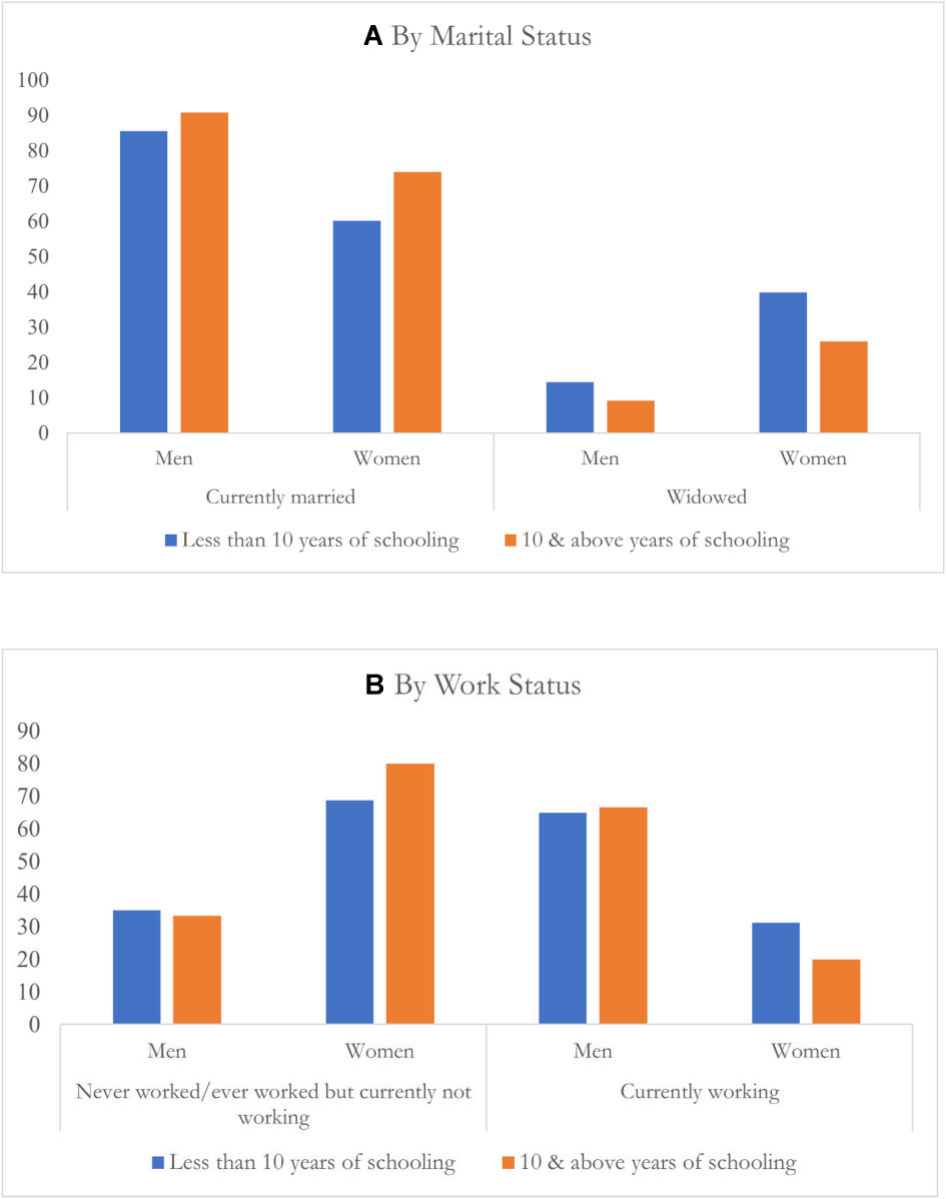
Underlying mechanisms of the gendered heterogeneous relationship between education and NCDs.

However, the contributions of marital status and working status are significantly different for men and women. For instance, marital status contributes positively in explaining the educational gap in NCDs (3.9%) and CVDs (3.2%) in men, while for women it contributes negatively (NCDs −11.7% and CVDs −15.8%). Similarly, working status positively contributes to the educational differences in the prevalence of NCDs (13.6%), CVDs (14.8%) and diabetes (8.7%) in women, while for men the contribution is not statistically significant. Supplementary Table S4 gives complete details of the decomposition analysis. To further investigate the contribution made by marital status and work status to the educational differences in NCDs, we plotted marital status and work status by gender and educational intersectional axes. The results, presented Figure 4, illustrate the contrasting distribution of the characteristics of marital and employment status of men and women, showing that differential marital and work status are the underlying mechanisms of the gendered heterogeneous relationship between education and NCDs.

## Discussion

The findings of this study demonstrate that the relationship between education and NCD reporting is sensitive to gender, exhibiting contrasting patterns between men and women. Among men, there is a linear positive association, indicating NCDs increase with an increase in education level. However, this pattern was the opposite for women: with an increase in level of education, the likelihood of having NCDs is reduced. The study also attempted to explain this contrasting pattern between education and NCDs for men and women using decomposition analyses. The findings from the decomposition analyses show a distinct role of marital status and working status of men and women in the gendered heterogeneous relationship of education and NCDs in India. An assessment of the distribution of marital status stratified by education level shows a greater occurrence of widowhood for women than men. Furthermore, a notable proportion of educated women are not participating in the workforce, in contrast to men. The contrasting distribution of the characteristics of marital and employment status for men and women could potentially provide a viable explanation for the gendered heterogeneous relationship of education and NCDs, as there is a greater probability of reporting NCDs among working and currently married individuals as compared with their counterparts. A greater prevalence of widowed and unemployed women may lead to less exposure and opportunities to detect and report NCDs. Therefore, the elimination of gender discrimination in marital norms and labour market inequalities is critical to diagnose and report NCDs in educated women.

Numerous previous studies have contended that self-reported measures of morbidities are misleading^34^ due to the absence of an observed strong negative association between education level and morbidity prevalence.^17,35,36^ Individual's assessment of their health is directly contingent on their social experience, so those who are socially disadvantaged will fail to perceive and report the presence of illness or health deficits.^37^

Despite the fact that the relationship between education and NCDs is well established, this study contributes additional perspective by highlighting the gendered heterogeneous relationship of education and NCDs. The gender-sensitive association of education and NCDs is attributed to the influence of marital and employment status on NCDs for men and women. A higher prevalence of widowed and unemployed among women may hinder their ability to diagnose and report NCDs. In India, a country with rigid gender norms and traditional kinship systems,^38,39^ widowhood is often regarded as a dreaded phase of life among some groups, particularly for women.^40^ Traditionally, in Indian culture, the social position of the woman is attached to the husband’s socio-economic situation, therefore losing a husband early pushes women into a precarious phase characterized by severe poverty, a lack of social support and the inability to remarry.^41^ Also, existing evidence suggests that the societal stigma and poor socio-economic status associated with widowhood significantly correlates with reduced healthcare utilization among women compared with men. Such stigma not only exacerbates the vulnerability to illness, but also hampers access to healthcare and diagnosis,^42,43^ causing poor reporting of illnesses,^44^ as supported by our findings.

Furthermore, the higher prevalence of unemployment among women also plays a significant role in the gendered heterogeneous relationship of education and NCDs. The role of working status in women's healthcare utilization and reporting of illness is crucial, as it encompasses various dimensions of financial independence, knowledge and autonomy. Women who are employed often have greater financial resources, which enables them to seek healthcare services without relying on others for financial support. This financial independence empowers working women to prioritize their health needs and access medical care promptly when necessary. Furthermore, working women tend to have more exposure to information about illnesses and healthcare practices through their work environments, which enhances their knowledge of health-related issues. This increased awareness can influence their ability to recognize symptoms, understand the importance of seeking medical attention and effectively communicate their health concerns to healthcare providers, thereby facilitating more accurate reporting of illness.^45^ Our findings align with these observations, that unemployment acts as a barrier to accessing health information about illness, thus contributing to a reporting bias of illness among women.^46^

## Conclusions and Policy Implications

In conclusion, our study found that the observed gendered heterogeneous pattern in the association of education and NCDs is deceptive due to self-reporting bias in morbidity. However, more coherent elucidations of the results are needed, especially on how to utilize these findings for health policy interventions. For instance, the decomposition analyses show the distinct role of marital status and working status of men and women in the gendered heterogeneous relationship of education and NCDs, which provides a number of intriguing insights. First, the bias in self-reported morbidity, especially among women, is primarily influenced by their work and marital status. Second, enhancing women's engagement in the workforce alongside education not only elevates their financial standing, but also enhances their social connections. With better financial stability and increased social interactions, women are more likely to be aware of their health conditions and have the means and support to diagnose, seek treatment and report any diseases they may experience. Third, being a widow acts as a deterrent to open communication and acknowledgment of health problems among women. This could be due to various reasons, including societal norms and a sense of financial vulnerability that may accompany widowhood. Also, it is important to acknowledge the potential impact of self-reporting bias in morbidity data while examining the relationship between education and NCDs. Providing economic independence can alleviate the sense of vulnerability and empower women to prioritize and address their health concerns. The study highlights the importance of intersecting social hierarchies (e.g. education and gender alongside marital status and working status) for explaining health inequities and illness reporting to provide strategies to improve health equity through healthcare policy.

## Supplementary Material

ihae037_Supplemental_Files

## Data Availability

This study utilized a secondary source of data that is freely available in the public domain by request (https://iipsindia.ac.in/sites/default/files/LASI_DataRequestForm_0.pdf).
