# Supplementary material for: Education and non-communicable diseases in India: an exploration of gendered heterogeneous relationships
Source: Int Health. 2024 May 24;17(2):168–78. doi: 10.1093/inthealth/ihae037 (PMC11879495; doi:10.1093/inthealth/ihae037)
Supplement: ihae037_Supplemental_Files [file ihae037_supplemental_files.zip › Supplementary Table S1.docx]

**Supplementary Table 1**: Socio-economic and demographic characteristics of the study participants by gender

| **Characteristics** | **Total population N= 65,257** | % | **Men N=30334** | % | **Women N=34,923** | % | **P Value** |
| --- | --- | --- | --- | --- | --- | --- | --- |
| **Age group** | | | | | | | |
| 45-49 | 13,114 | 18.9 | 5,880 | 17.6 | 7,234 | 20 | 0.000 |
| 50-54 | 10,861 | 16.1 | 4,984 | 16.5 | 5,877 | 15.8 |  |
| 55-59 | 9,970 | 14.9 | 4,450 | 14.3 | 5,520 | 15.4 |  |
| 60 and above | 31,312 | 50.1 | 15,020 | 51.7 | 16,292 | 48.8 |  |
| **Place of residence** | | | | | | | |
| Rural | 42,264 | 68.7 | 19,823 | 70.00 | 22,441 | 67.6 | 0.004 |
| Urban | 22,993 | 31.3 | 10,511 | 30.00 | 12,482 | 32.4 |  |
| **Religion** | | | | | | | |
| Hindu | 47,905 | 82.2 | 22,357 | 82.5 | 25,548 | 81.9 | 0.128 |
| Muslim | 7,743 | 11.3 | 3,521 | 11.3 | 4,222 | 11.3 |  |
| Christian | 6,501 | 3 | 2,984 | 2.6 | 3,517 | 3.4 |  |
| Others* | 3,108 | 3.5 | 1,472 | 3.6 | 1,636 | 3.4 |  |
| **Caste** | | | | | | | |
| Scheduled Caste (SC) | 10,931 | 19.2 | 5,032 | 19 | 5,899 | 19.4 | 0.599 |
| Scheduled Tribe (ST) | 11,336 | 8.6 | 5,243 | 8.5 | 6,093 | 8.7 |  |
| Other Backward Class (OBC) | 24,552 | 45.4 | 11,479 | 45.6 | 13,073 | 45.3 |  |
| Others | 18,438 | 26.7 | 8,580 | 26.9 | 9,858 | 26.6 |  |
| **Level of education** | | | | | | | |
| No education | 30,687 | 50.75 | 9,443 | 33.97 | 21,244 | 64.94 | 0.000 |
| Less than 5 years of schooling | 7,440 | 10.92 | 4,042 | 13.34 | 3,398 | 8.87 |  |
| 5-9 years of schooling | 14,803 | 20.45 | 8,542 | 26.38 | 6,261 | 15.44 |  |
| 10 and above years of schooling | 12,327 | 17.88 | 8,307 | 26.3 | 4,020 | 10.76 |  |
| **Marital status** | | | | | | | |
| Currently married | 48,557 | 73.3 | 26,583 | 87 | 21,974 | 61.7 | 0.000 |
| Widowed | 14,513 | 23.8 | 2,759 | 10.2 | 11,754 | 35.3 |  |
| Others ** | 2,187 | 2.9 | 992 | 2.8 | 1,195 | 3 |  |
| **Living arrangement** | | | | | | | |
| Living alone | 2,295 | 3.7 | 587 | 1.8 | 1,708 | 5.3 | 0.000 |
| Living with spouse, children and/or others | 47,667 | 72.3 | 26,222 | 86.3 | 21,445 | 60.4 |  |
| Living with children and/or others | 15,295 | 24.1 | 3,525 | 12 | 11,770 | 34.3 |  |
| **Working status** | | | | | | | |
| Currently working | 30,053 | 26.1 | 19,712 | 65.3 | 10,341 | 30.1 | 0.000 |
| Worked in the past but currently not working | 17,291 | 46.2 | 9,382 | 31.6 | 7,909 | 24.3 |  |
| Never worked | 17,913 | 27.6 | 1,240 | 3.1 | 16,673 | 45.6 |  |
| **MPCE Quintile** | | | | | | | |
| Poorest | 12,879 | 20.9 | 5,933 | 20.6 | 6,946 | 21.2 | 0.474 |
| Poor | 13,129 | 21.3 | 6,059 | 21.3 | 7,070 | 21.3 |  |
| Middle | 13,109 | 20.3 | 6,079 | 20.2 | 7,030 | 20.5 |  |
| Richer | 13,156 | 19.5 | 6,180 | 19.5 | 6,976 | 19.5 |  |
| Richest | 12,984 | 18 | 6,083 | 18.5 | 6,901 | 17.6 |  |
| **Region** | | | | | | | |
| Northern | 11,913 | 12.3 | 5,521 | 12.2 | 6,392 | 12.4 | 0.000 |
| Central | 8,899 | 20.7 | 4,301 | 22 | 4,598 | 19.5 |  |
| Eastern | 11,543 | 23.2 | 5,456 | 23.9 | 6,087 | 22.7 |  |
| North-eastern | 8,476 | 3.5 | 4,059 | 3.5 | 4,417 | 3.4 |  |
| Western | 8,824 | 16.5 | 4,011 | 16 | 4,813 | 17 |  |
| Southern | 15,602 | 23.8 | 6,986 | 22.4 | 8,616 | 25.1 |  |

**Note:** p values were generated from χ2 test

Religion; others*: Sikh, Buddhist/neo-Buddhist, Jain, Jewish and Parsi/Zoroastrian

Marital status; others**: Never married/divorced/separated/live-in-relationship
