# Supplementary material for: Education and non-communicable diseases in India: an exploration of gendered heterogeneous relationships
Source: Int Health. 2024 May 24;17(2):168–78. doi: 10.1093/inthealth/ihae037 (PMC11879495; doi:10.1093/inthealth/ihae037)
Supplement: ihae037_Supplemental_Files [file ihae037_supplemental_files.zip › Supplementary Table S2.docx]

**Supplementary Table S2:**  Prevalence of NCDs, CVDs and Diabetes among men and women older adults in India by level of education in India,2017-18

| Level of education | NCDs | | | | CVDs | | | | Diabetes | | | | |
| --- | --- | --- | --- | --- | --- | --- | --- | --- | --- | --- | --- | --- | --- |
|  | **Men** | | **Women** | | **Men** | | **Women** | | **Men** | | **Women** | | |
|  | **Adjusted prevalence** | **Unadjusted prevalence** | **Adjusted prevalence** | **Unadjusted prevalence** | **Adjusted prevalence** | **Unadjusted prevalence** | **Adjusted prevalence** | **Unadjusted prevalence** | **Adjusted prevalence** | **Unadjusted prevalence** | **Adjusted prevalence** | **Unadjusted prevalence** |  |
| No education | 36.2 | 38.2 | 43.1 | 45.1 | 20.3 | 21.7 | 27.4 | 28.6 | 6 | 7.6 | 6.7 | 8 |  |
| Less than 5 years of schooling | 41.6 | 43.1 | 52.8 | 58.2 | 23.7 | 24.5 | 34.6 | 37.6 | 9.5 | 10.5 | 11.1 | 16.1 |  |
| 5-9 years of schooling | 43.9 | 43.6 | 51.7 | 56.6 | 27.3 | 27.4 | 34.6 | 39.9 | 13.1. | 12.2 | 11.1 | 18.0 |  |
| 10 & above years of schooling | 51.7 | 49.2 | 51.1 | 56.1 | 35.6 | 33.1 | 34.1 | 40.3 | 20.2 | 20.1 | 11.0 | 23.2 |  |

**Note:** Prevalence were adjusted for different socio-economic factors (Age-group, residence, religion, caste, marital status, working status, living-arrangement, and Monthly per capita expenditure (MPCE)
