# Supplementary material for: Education and non-communicable diseases in India: an exploration of gendered heterogeneous relationships
Source: Int Health. 2024 May 24;17(2):168–78. doi: 10.1093/inthealth/ihae037 (PMC11879495; doi:10.1093/inthealth/ihae037)
Supplement: ihae037_Supplemental_Files [file ihae037_supplemental_files.zip › Supplementary Table S3.docx]

**Supplementary Table S3 (A):** Logistic regression model describing the association between prevalence of NCDs and education level along with other socio-economic variables among older adults by gender in India,2017-18

| Background characteristics | Men | | | Women | |
| --- | --- | --- | --- | --- | --- |
|  | **Odds ratio** | | **95% CI** | **Odds ratio** | **95% CI** |
| Education level | | | | | |
| No education® | | | | | |
| Less than 5 years of schooling | 1.18*** | [1.09,1.28] | | 1.46*** | [1.34,1.57] |
| 5-9 years of schooling | 1.26*** | [1.18,1.35] | | 1.35*** | [1.26,1.44] |
| 10 and above years of schooling | 1.43*** | [1.33,1.53] | | 1.20*** | [1.11,1.31] |
| Age-group | | | | | |
| 45-49® | | | | | |
| 50-54 | 1.45*** | [1.33,1.57] | | 1.39*** | [1.29,1.50] |
| 55-59 | 1.73*** | [1.59,1.89] | | 1.70*** | [1.56,1.83] |
| 60 and above | 2.17*** | [2.01,2.34] | | 2.09*** | [1.95,2.23] |
| Place of residence | | | | | |
| Rural® | | | | | |
| Urban | 1.46*** | [1.38,1.54] | | 1.47*** | [1.40,1.55] |
| Religion | | | | | |
| Hindu® | | | | | |
| Muslim | 1.23*** | [1.14,1.33] | | 1.49*** | [1.38,1.60] |
| Christian | 0.97 | [0.87,1.08] | | 1.11* | [1.01,1.22] |
| Others* | 1.29*** | [1.14,1.45] | | 1.30*** | [1.16,1.45] |
| Caste | | | | | |
| Scheduled caste/Tribe (SC/ST) ® | | | | | |
| Other backward class (OBC) | 1.18*** | [1.10,1.25] | | 1.20*** | [1.14,1.27] |
| Others | 1.25*** | [1.16,1.34] | | 1.31*** | [1.23,1.39] |
| Marital Status | | | | | |
| Currently married® | | | | | |
| Widowed | 0.95 | [0.74,1.20] | | 1.36** | [1.13,1.65] |
| Others** | 0.85 | [0.65,1.10] | | 1.18 | [0.94,1.46] |
| Living arrangement | | | | | |
| Living alone® | | | | | |
| Living with spouse & children | 1.38* | [1.06,1.78] | | 1.35** | [1.10,1.66] |
| Living with children & others | 1.21 | [0.99,1.47] | | 1.16** | [1.04,1.29] |
| Working status | | | | | |
| Never worked® | | | | | |
| Currently working | 0.70*** | [0.62,0.79] | | 0.71*** | [0.67,0.75] |
| Worked in past but currently _notworking | 1.40*** | [1.24,1.59] | | 1.18*** | [1.11,1.25] |
| Monthly per capita expenditure (MPCE) | | | | | |
| Poorest® | | | | | |
| Poorer | 1.24*** | [1.14,1.34] | | 1.20*** | [1.12,1.29] |
| Middle | 1.33*** | [1.23,1.44] | | 1.38*** | [1.28,1.48] |
| Richer | 1.61*** | [1.48,1.74] | | 1.55*** | [1.44,1.66] |
| Richest | 1.91*** | [1.76,2.08] | | 1.82*** | [1.69,1.96] |
| Region | | | | | |
| North® | | | | | |
| Central | 0.64*** | [0.58,0.69] | | 0.59*** | [0.54,0.64] |
| East | 0.97 | [0.89,1.05] | | 0.87*** | [0.81,0.94] |
| Northeast | 0.76*** | [0.68,0.84] | | 0.66*** | [0.60,0.72] |
| West | 1.07 | [0.98,1.17] | | 0.98 | [0.91,1.07] |
| South | 1.41*** | [1.30,1.53] | | 1.28*** | [1.18,1.37] |

**Supplementary Table S3 (B)**: Logistic regression model describing the association between prevalence of CVDs and education level along with other socio-economic variables among older adults by gender in India,2017-18

| **Background characteristics** | **Men** | | **Women** | |
| --- | --- | --- | --- | --- |
|  | **Odds ratio** | **95% CI** | **Odds ratio** | **95% CI** |
| **Education level** | | | | |
| No education® | | | | |
| Less than 5 years of schooling | 1.15** | [1.05,1.26] | 1.42*** | [1.31,1.53] |
| 5-9 years of schooling | 1.36*** | [1.26,1.46] | 1.36*** | [1.27,1.46] |
| 10 & above years of schooling | 1.68*** | [1.55,1.82] | 1.16*** | [1.07,1.26] |
| **Age-group** | | | | |
| 45-49® | | | | |
| 50-54 | 1.40*** | [1.27,1.55] | 1.35*** | [1.24,1.46] |
| 55-59 | 1.75*** | [1.59,1.93] | 1.65*** | [1.52,1.79] |
| 60 and above | 2.11*** | [1.93,2.30] | 2.12*** | [1.98,2.28] |
| **Place of residence** | | | | |
| Rural® | | | | |
| Urban | 1.52*** | [1.43,1.61] | 1.49*** | [1.42,1.57] |
| **Religion** | | | | |
| Hindu® | | | | |
| Muslim | 1.2*** | [1.12,1.33] | 1.54*** | [1.43,1.65] |
| Christian | 1.07 | [0.95,1.20] | 1.19*** | [1.08,1.31] |
| Others* | 1.40*** | [1.24,1.59] | 1.39*** | [1.24,1.55] |
| **Caste** | | | | |
| Scheduled caste/tribe (SC/ST) ® | | | | |
| Other backward class (OBC) | 1.10** | [1.03,1.18] | 1.20*** | [1.13,1.27] |
| Others | 1.25*** | [1.16,1.35] | 1.31*** | [1.23,1.40] |
| **Marital Status** | | | | |
| Currently married® | | | | |
| Widowed | 0.91 | [0.70,1.19] | 1.38** | [1.13,1.69] |
| Others** | 0.78 | [0.59,1.05] | 1.11 | [0.88,1.41] |
| **Living arrangement** | | | | |
| Living alone® | | | | |
| Living with spouse & children | 1.17 | [0.88,1.55] | 1.30* | [1.04,1.62] |
| Living with children & others | 1.15 | [0.93,1.42] | 1.18** | [1.06,1.32] |
| **Working status** | | | | |
| Never worked® | | | | |
| Currently working | 0.66*** | [0.58,0.75] | 0.68*** | [0.64,0.73] |
| Worked in past but currently _notworking | 1.26*** | [1.10,1.44] | 1.05 | [0.98,1.11] |
| **Monthly per capita expenditure (MPCE)** | | | | |
| Poorest® | | | | |
| Poorer | 1.17*** | [1.07,1.28] | 1.18*** | [1.09,1.27] |
| Middle | 1.28*** | [1.17,1.40] | 1.36*** | [1.26,1.47] |
| Richer | 1.50*** | [1.37,1.64] | 1.54*** | [1.42,1.66] |
| Richest | 1.75*** | [1.60,1.92] | 1.72*** | [1.59,1.86] |
| **Region** | | | | |
| North® | | | | |
| Central | 0.60*** | [0.54,0.67] | 0.59*** | [0.54,0.65] |
| East | 0.91* | [0.83,1.01] | 0.76*** | [0.69,0.82] |
| Northeast | 0.96 | [0.86,1.07] | 0.78*** | [0.71,0.86] |
| West | 0.91 | [0.83,1.00] | 0.76*** | [0.69,0.83] |
| South | 1.18*** | [1.08,1.28] | 0.91** | [0.84,0.98] |

**Supplementary Table S3 (C):** Logistic regression model describing the association between prevalence of Diabetes and education level along with other socio-economic variables among older adults by gender in India,2017-18

| **Background characteristics** | **Men** | | **Women** | |
| --- | --- | --- | --- | --- |
|  | **Odds ratio** | **95% CI** | **Odds ratio** | **95% CI** |
| **Education level** | | | | |
| No education® | | | | |
| Less than 5 years of schooling | 1.40*** | [1.23,1.60] | 1.62*** | [1.45,1.81] |
| 5-9 years of schooling | 1.79*** | [1.62,1.99] | 1.51*** | [1.38,1.66] |
| 10 & above years of schooling | 2.45*** | [2.20,2.73] | 1.54*** | [1.38,1.72] |
| **Age-group** | | | | |
| 45-49® | | | | |
| 50-54 | 1.52*** | [1.33,1.73] | 1.54*** | [1.36,1.75] |
| 55-59 | 1.69*** | [1.47,1.93] | 2.19*** | [1.95,2.48] |
| 60 and above | 2.03*** | [1.80,2.29] | 2.29*** | [2.05,2.55] |
| **Place of residence** | | | | |
| Rural® | | | | |
| Urban | 2.02*** | [1.87,2.17] | 1.95*** | [1.81,2.10] |
| **Religion** | | | | |
| Hindu® | | | | |
| Muslim | 1.21*** | [1.09,1.36] | 1.34*** | [1.21,1.47] |
| Christian | 1.18* | [1.02,1.37] | 1.41*** | [1.24,1.60] |
| Others* | 1.39*** | [1.17,1.64] | 1.27** | [1.08,1.49] |
| **Caste** | | | | |
| Scheduled caste/tribe (SC/ST) | | | | |
| Other backward class (OBC) | 1.20*** | [1.07,1.32] | 1.16** | [1.05,1.27] |
| Others | 1.24*** | [1.12,1.37] | 1.31*** | [1.19,1.45] |
| **Marital Status** | | | | |
| Currently married® | | | | |
| Widowed | 0.83 | [0.59,1.17] | 1.69** | [1.20,2.41] |
| Others** | 0.71 | [0.48,1.03] | 1.59* | [1.08,2.33] |
| **Living arrangement** | | | | |
| Living alone® | | | | |
| Living with spouse & children | 1.35 | [0.92,1.98] | 2.09*** | [1.44,3.04] |
| Living with children & others | 1.26 | [0.93,1.70] | 1.14 | [0.91,1.35] |
| **Working status** | | | | |
| Never worked® | | | | |
| Currently working | 0.71** | [0.59,0.85] | 0.53*** | [0.48,0.58] |
| Worked in past but currently _not working | 1.19 | [0.99,1.43] | 0.96 | [0.89,1.05] |
| **Monthly per capita expenditure (MPCE)** | | | | |
| Poorest® | | | | |
| Poorer | 1.31*** | [1.15,1.48] | 0.99 | [0.88,1.11] |
| Middle | 1.39*** | [1.23,1.58] | 1.25*** | [1.12,1.40] |
| Richer | 1.69*** | [1.49,1.91] | 1.41*** | [1.26,1.58] |
| Richest | 2.02*** | [1.79,2.29] | 1.66*** | [1.48,1.85] |
| **Region** | | | | |
| North® | | | | |
| Central | 0.94 | [0.81,1.09] | 0.67*** | [0.58,0.78] |
| East | 1.24*** | [1.09,1.41] | 0.88* | [0.78,0.99] |
| Northeast | 0.88 | [0.74,1.03] | 0.68*** | [0.58,0.79] |
| West | 1.49*** | [1.36,1.69] | 1.18** | [1.05,1.33] |
| South | 2.19*** | [1.96,2.46] | 1.88** | [1.69,2.09] |

**Note;** * p<0.05, ** p<0.01, *** p<0.001; 1.00=reference category

Religion; other*: Sikh, Buddhist/neo-Buddhist, Jain, Jewish and Parsi/Zoroastrian

Marital status; others**: Never married/divorced/separated/live-in-relationship
