# Supplementary material for: Education and non-communicable diseases in India: an exploration of gendered heterogeneous relationships
Source: Int Health. 2024 May 24;17(2):168–78. doi: 10.1093/inthealth/ihae037 (PMC11879495; doi:10.1093/inthealth/ihae037)
Supplement: ihae037_Supplemental_Files [file ihae037_supplemental_files.zip › Supplementary Table S4.docx]

**Supplementary Table S4A):** Oaxaca decomposition: Contribution of selected predictors on the prevalence differences of NCDs between educated (10 and above years of schooling) and non-educated (less than 10 years schooling) men older adults in India,2017-18

| Summary of Oaxaca decomposition | | | | |
| --- | --- | --- | --- | --- |
| NCDs (Men) | | | | |
| NCDs | **Coefficient** | **Standard error** | **95% confidence interval** | |
| 10 & above years of schooling | 0.502*** | 0.006*** | 0.491 | 0.513 |
| Less than 10 years of schooling | 0.397*** | 0.003*** | 0.391 | 0.404 |
| Difference | 0.105*** | 0.007*** | 0.092 | 0.118 |
| Explained | 0.058*** | 0.003*** | 0.051 | 0.064 |
| Unexplained | 0.047*** | 0.007*** | 0.034 | 0.061 |
| % explained | 55.4 | | | |
| % unexplained (residual) | 44.6 | | | |
| Details of explained part Explanatory factors | | **% contribution of the total difference** | **Standard error** | |
| Age-group | | -16.8*** | 0.001 | |
| Place of residence | | 55.0*** | 0.002 | |
| Living arrangement | | 0.1 | 0.000 | |
| Religion | | -2.9*** | 0.000 | |
| Caste | | 16.7*** | 0.001 | |
| Marital-Status | | 3.9*** | 0.000 | |
| Working-Status | | 3.2 | 0.001 | |
| MPCE-Quintile | | 37.6*** | 0.001 | |
| Region | | 3.0*** | 0.000 | |
| Total explained part | 100 | | | |

**Note:** * p<0.05, ** p<0.01, *** p<0.001

**Supplementary Table S4(B):** Oaxaca decomposition: Contribution of selected predictors on the prevalence differences of NCDs between educated (10 and above years of schooling) and non-educated (less than 10 years schooling) women older adults in India,2017-18

| Summary of Oaxaca decomposition | | | | |
| --- | --- | --- | --- | --- |
| NCDs (Women) | | | | |
| NCDs | **Coefficient** | **Standard error** | **95% confidence interval** | |
| 10 and above years of schooling | 0.563*** | 0.008 | 0.548 | 0.578 |
| Less than 10 years of schooling | 0.482*** | 0.003 | 0.477 | 0.488 |
| Difference | 0.081*** | 0.008 | 0.064 | 0.097 |
| Explained | 0.068*** | 0.004 | 0.060 | 0.076 |
| Unexplained | 0.013 | 0.009 | -0.004 | 0.030 |
| % explained | 84.0 | | | |
| % unexplained (residual) | 16.0 | | | |
| Details of explained part Explanatory factors | | **% contribution of the total difference** | **Standard error** | |
| Age-group | | -28.8*** | 0.001 | |
| Place of residence | | 71.5*** | 0.003 | |
| Living arrangement | | 0.7 | 0.000 | |
| Religion | | -5.4*** | 0.001 | |
| Caste | | 18.6*** | 0.001 | |
| Marital-Status | | -11.7*** | 0.001 | |
| Working-Status | | 13.6*** | 0.001 | |
| MPCE-Quintile | | 40.9*** | 0.002 | |
| Region | | 0.6 | 0.001 | |
| Total explained part | 100 | | | |

**Note:** * p<0.05, ** p<0.01, *** p<0.001

**Supplementary Table S4(C):** Oaxaca decomposition: Contribution of selected predictors on the prevalence differences of CVDs between educated (10 and above years of schooling) and non-educated (less than 10 years schooling) men older adults in India,2017-18

| Summary of Oaxaca decomposition | | | | |
| --- | --- | --- | --- | --- |
| CVD (Men) | | | | |
| CVD | **Coefficient** | **Std. Err.** | **95% Confidence interval** | |
| 10 and above years of schooling | 0.354*** | 0.005 | 0.343 | 0.364 |
| Less than 10 years of schooling | 0.237*** | 0.003 | 0.231 | 0.243 |
| Difference | 0.117*** | 0.006 | 0.105 | 0.129 |
| Explained | 0.045*** | 0.003 | 0.040 | 0.051 |
| Unexplained | 0.071*** | 0.006 | 0.059 | 0.084 |
| % Explained | 38.8 | | | |
| % Unexplained (residual) | 61.2 | | | |
| Details of explained part Explanatory factors | | **% Contribution of the total difference** | **Standard error** | |
| Age-group | | -16.2*** | 0.001 | |
| Place of residence | | 63.8*** | 0.002 | |
| Living-arrangement | | 0.1 | 0.000 | |
| Religion | | -2.9*** | 0.000 | |
| Caste | | 13.2*** | 0.001 | |
| Marital-Status | | 3.2*** | 0.000 | |
| Working-Status | | 3.4* | 0.001 | |
| MPCE-Quintile | | 35.3*** | 0.001 | |
| Region | | 0.2 | 0.000 | |
| Total explained part | 100 | | | |

**Note:** * p<0.05, ** p<0.01, *** p<0.001

**Supplementary Table S4(D**): Oaxaca decomposition: Contribution of selected predictors on the prevalence differences of CVDs between educated (10 and above years of schooling) and non-educated (less than 10 years schooling) women older adults in India,2017-18

| Summary of Oaxaca decomposition | | | | |
| --- | --- | --- | --- | --- |
| CVDs (Women) | | | | |
| CVDs | **Coefficient** | **Std. Err.** | **95% Conf interval** | |
| 10 and above years of schooling | 0.382*** | 0.008 | 0.367 | 0.398 |
| Less than 10 years of schooling | 0.326*** | 0.003 | 0.320 | 0.331 |
| Difference | 0.057*** | 0.008 | 0.040 | 0.073 |
| Explained | 0.054*** | 0.004 | 0.047 | 0.062 |
| Unexplained | 0.003 | 0.009 | -0.015 | 0.020 |
| % Explained | 95.6 | | | |
| % Unexplained (residual) | 4.4 | | | |
| Details of explained part Explanatory factors | | **% Contribution of the total difference** | **Standard error** | |
| Age-group | | -35.8*** | 0.001 | |
| Place of residence | | 81.0*** | 0.003 | |
| Living-arrangement | | 1.5 | 0.000 | |
| Religion | | -8.2*** | 0.001 | |
| Caste | | 19.2*** | 0.001 | |
| Marital-Status | | -15.8*** | 0.001 | |
| Working-Status | | 14.8*** | 0.001 | |
| MPCE-Quintile | | 43.2*** | 0.002 | |
| Region | | 0.1 | 0.000 | |
| Total explained part | 100 | | | |

**Note:** * p<0.05, ** p<0.01, *** p<0.001

**Supplementary Table S4(E):** Oaxaca decomposition: Contribution of selected predictors on the prevalence differences of Diabetes between educated (10 and above years of schooling) and non-educated (less than 10 years schooling) men older adults in India,2017-18

| Summary of Oaxaca decomposition | | | | |
| --- | --- | --- | --- | --- |
| Diabetes (Men) | | | | |
| Diabetes | **Coefficient** | **Std. Err.** | **95% Conf interval** | |
| 10 and above years of schooling | 0.213*** | 0.005 | 0.204 | 0.222 |
| Less than 10 years of schooling | 0.102*** | 0.002 | 0.098 | 0.106 |
| Difference | 0.111*** | 0.005 | 0.101 | 0.121 |
| Explained | 0.043*** | 0.002 | 0.038 | 0.047 |
| Unexplained | 0.069*** | 0.005 | 0.058 | 0.079 |
| % Explained | 38.2 | | | |
| % Unexplained (residual) | 61.8 | | | |
| Details of explained part Explanatory factors | | **% Contribution of the total difference** | **Standard error** | |
| Age-group | | -8.6*** | 0.000 | |
| Place of residence | | 68.0*** | 0.002 | |
| Living-arrangement | | 0.1 | 0.000 | |
| Religion | | -1.2 | 0.000 | |
| Caste | | 8.7*** | 0.001 | |
| Marital-Status | | 4.0*** | 0.000 | |
| Working-Status | | 1.7 | 0.000 | |
| MPCE-Quintile | | 25.4*** | 0.001 | |
| Region | | 1.9*** | 0.000 | |
| Total explained part | | 100 |  | |

**Note:** * p<0.05, ** p<0.01, *** p<0.001

**Supplementary** **Table S4(F):** Oaxaca decomposition: Contribution of selected predictors on the prevalence differences of Diabetes between educated (10 and above years of schooling) and non-educated (less than 10 years schooling) women older adults in India,2017-18

| Summary of Oaxaca decomposition | | | | |
| --- | --- | --- | --- | --- |
| Diabetes (Women) | | | | |
| Diabetes | **Coefficient** | **Std. Err.** | **95% Conf interval** | |
| 10 and above years of schooling | 0.209*** | 0.007 | 0.196 | 0.222 |
| Less than 10 years of schooling | 0.113*** | 0.002 | 0.110 | 0.117 |
| Difference | 0.095*** | 0.007 | 0.082 | 0.109 |
| Explained | 0.053*** | 0.003 | 0.048 | 0.058 |
| Unexplained | 0.042*** | 0.007 | 0.028 | 0.057 |
| % Explained | 55.7 | | | |
| % Unexplained (residual) | 44.3 | | | |
| Details of explained part Explanatory factors | | **% Contribution of the total difference** | **Standard error** | |
| Age-group | | -14.4*** | 0.001 | |
| Place of residence | | 75.9*** | 0.002 | |
| Living-arrangement | | 0.1 | 0.000 | |
| Religion | | -2.7*** | 0.000 | |
| Caste | | 7.6*** | 0.001 | |
| Marital-Status | | 1.7 | 0.001 | |
| Working-Status | | 8.7*** | 0.000 | |
| MPCE-Quintile | | 22.9*** | 0.001 | |
| Region | | 0.2 | 0.000 | |
| Total explained part | 100 | | | |

**Note:** * p<0.05, ** p<0.01, *** p<0.001
